# Production of human pro-relaxin H2 in the yeast *Pichia pastoris*

**DOI:** 10.1186/s12896-016-0319-0

**Published:** 2017-01-14

**Authors:** D. Cimini, K. Della Corte, R. Finamore, L. Andreozzi, A. Stellavato, A. V. A. Pirozzi, F. Ferrara, R. Formisano, M. De Rosa, M. Chino, L. Lista, A. Lombardi, V. Pavone, C. Schiraldi

**Affiliations:** 1 Department of Experimental Medicine, Section of Biotechnology and Molecular Biology, Second University of Naples and University of Campania Luigi Vanvitelli, via de Crecchio 7, 80138 Naples, Italy; 2 Department of Chemical Sciences, University of Naples Federico II, Via Cintia I, 80126 Naples, Italy

**Keywords:** Pro-relaxin, Pro-hormone, Protein instability, *Pichia pastoris*, IMAC, LCMS

## Abstract

**Background:**

Initially known as the reproductive hormone, relaxin was shown to possess other therapeutically useful properties that include extracellular matrix remodeling, anti-inflammatory, anti-ischemic and angiogenic effects. All these findings make relaxin a potential drug for diverse medical applications. Its precursor, pro-relaxin, is an 18 kDa protein, that shows activity in *in vitro* assays. Since extraction of relaxin from animal tissues raises several issues, prokaryotes and eukaryotes were both used as expression systems for recombinant relaxin production. Most productive results were obtained when using *Escherichia coli* as a host for human relaxin expression. However, in such host, relaxin precipitated in the form of inclusion bodies and, therefore, required several expensive recovery steps as cell lysis, refolding and reduction.

**Results:**

To overcome the issues related to prokaryotic expression here we report the production and purification of secreted human pro-relaxin H2 by using the methylotrophic yeast *Pichia pastoris* as expression host. The methanol inducible promoter AOX1 was used to drive expression of the native and histidine tagged forms of pro-relaxin H2 in dual phase fed-batch experiments on the 22 L scale. Both protein forms presented the correct structure, as determined by mass spectrometry and western blotting analyses, and demonstrated to be biologically active in immune enzymatic assays. The presence of the tag allowed to simplify pro-relaxin purification obtaining higher purity.

**Conclusions:**

This work presents a strategy for microbial production of recombinant human pro-relaxin H2 in *Pichia pastoris* that allowed the obtainment of biologically active pro-hormone, with a final concentration in the fermentation broth ranging between 10 and 14 mg/L of product, as determined by densitometric analyses.

**Electronic supplementary material:**

The online version of this article (doi:10.1186/s12896-016-0319-0) contains supplementary material, which is available to authorized users.

## Background

Relaxin is a multifunctional peptide hormone initially identified in reproductive tissues during pregnancy and later classified as a pleiotropic factor that induces biochemical changes also in a number of non-reproductive organs. Indeed, relaxin receptors have also been found in brain, heart, skin, small intestine and blood vessels [1]. Three relaxin genes are present in the human genome and they code for the peptides indicated as H1, H2 and H3, which differ in their primary and tertiary structure, activity and expression site. H2, is the major circulating form and it is mainly produced in the corpus luteum [2]. Besides its well-established biological role during pregnancy in reproductive tissues, where it relaxes the pelvic ligaments and tendons, more recently relaxin has been shown to be involved in the maternal adaptation of the renal and cardiovascular systems. Moreover, the latest studies have also established the potential of relaxin as vasodilator, and its contribution in angiogenesis and extracellular-matrix remodeling [3–7]. Most of these functions are important in the underlying pathology of acute heart failure (AHF), in fact, cardio-renal protective effects along with a significant benefit of dyspnea relief in patients with this pathology seem to be attributable to the capacity of relaxin to improve systemic, cardiac and renal hemodynamics, and protect cells and organs from damage via its neuro-hormonal, anti-inflammatory, anti-remodeling, anti-fibrotic, anti-ischemic, and pro-angiogenic effects [3, 8]. In 2012 serelaxin, recombinant human relaxin, passed Phase III clinical trials for the treatment of AHF [9] and was appointed as “Breakthrough Therapy” by the US Food and Drug Administration [8]. Furthermore, by using an *in vivo* mouse model Hampel and colleagues found a novel role for Relaxin 2 for the maintenance and regeneration of the ocular epithelial cell layer, underlying a new potential therapeutic application in ophthalmology for the treatment of corneal ulcerations [6].

Differently from other peptide hormones belonging to the same family, such as pro-insulin, it was demonstrated that also the immature/unprocessed hormone pro-form, pro-relaxin, is biologically active [10, 11]. Although animal tissues represent a source of relaxin, their pro-relaxin content is very low. Moreover, extraction procedures are inefficient. About 0.03 mg of relaxin and 0.8 mg of pro-relaxin were obtained by using 165 g of placenta homogenate extracts from 40 hamsters [12] and a yield of 0.72 mg of pro-relaxin per gram of wet tissue was obtained from decapsulated corpus luteum of pregnant gilts [13]. In addition the relatively low structure similarity between animal derived H2 forms and human ones (eg. only share 55% identity) might elicit strong immunogenic responses thus reducing the field of application. Therefore, it is desirable to replace such animal-tissue extraction procedures with biotechnological processes, in order to generate larger and safer stocks of relaxin/pro-relaxin for therapeutic applications. The production of human relaxin/pro-relaxin by intensive cultures of genetically engineered microorganisms is more compatible with pharmaceutical applications. To date, different prokaryotic and eukaryotic hosts have been adopted to produce recombinant relaxin and pro-relaxin all sharing the same limitation represented by quite low expression levels [10, 11, 13–16]. However, human recombinant relaxin H2 was expressed in *E.coli* cells, purified from inclusion bodies and characterized, demonstrating biologically active in *in vitro* assays with human endometrial cells [17], and recently reached phase III clinical trials for patients with acute decompensated heart failure.

The theoretical possibility of secreting high levels of correctly folded human proteins in the yeast *P. pastoris,* and the availability of commercial protease-deficient strains that reduce protein degradation over time, encouraged our efforts towards the use of this yeast as host for the production of human pro-relaxin H2.

In the present work a simple approach was established for the production of recombinant human pro-relaxin H2 on a semi-defined medium exploiting glycerol/methanol fed-batch fermentations that result in the production of active pro-hormone as established by bioactivity assays.

## Results

### Strategy and construction of the recombinant strains

Since one of the main problems related to protein production and purification processes is degradation by endogenous proteases, the production of human pro-relaxin H2 was evaluated in a commercial *Pichia* knock-out strain that produces a lower number of vacuolar proteases. The aminoacid and the codon-optimized nucleotide sequences of the native and histidine tagged human pro-relaxin H2 used in this study are shown in Additional file 1. The pro-relaxin sequences with and without histidine tag were cloned in the pPink vector (Invitrogen) in frame with the Mat-α signal sequence to enable protein secretion. The histidine tag was fused to the C-terminus of the protein in order to compare different purification strategies. All constructs were sequence verified before integration into the genome of competent *Pichia* pink cells. The colonies that showed a change in color from pink to white were further analyzed, confirming the specific insertion of the pro-relaxin gene into their genome and generating the recombinant strains indicated in the following as *Pichia* pink-prel and *Pichia* pink-prelHis, respectively.

### Shakeflask and fermentation experiments

Eight positive colonies for each recombinant strain were screened in shakeflasks for pro-relaxin productivity by methanol induction. The supernatants of all clones collected after 72 h of methanol induction were concentrated and analyzed by western blotting. All colonies showed a band of the expected molecular weight. The colonies producing most intense bands were selected for further experiments (data not shown).

**Fig. 1:**
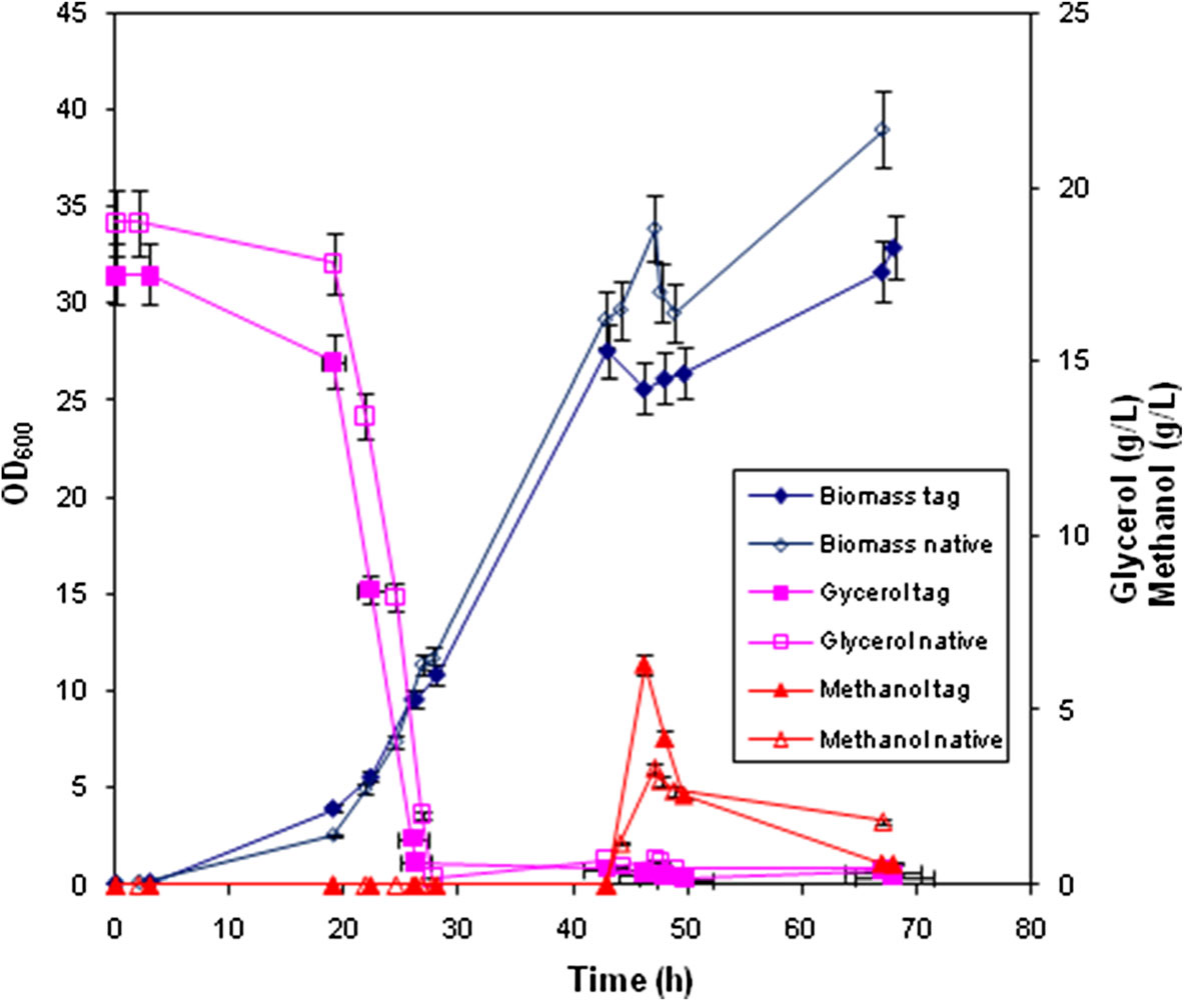
Dual phase fed-batch experiments. Time course of glycerol and methanol consumption, and biomass production during the processes. Native indicates parameters relative to the fermentation of the strain *Pichia* pink-prel that produces un-tagged pro-relaxin, whereas tagged refers to strain *Pichia* pink-prelHis producing His tagged pro-relaxin. Fermentation experiments were run in triplicate for each strain and the reported data are the mean values ± S.D

*Pichia* pink-prel and *Pichia* pink-prelHis were grown in fed-batch cultures involving initial batch and DO-stat growth on glycerol to increase biomass, followed by a switch to a methanol fed-batch phase to induce protein expression. After the batch phase about 300 g of total glycerol were fed to the cultures until a concentration of about 30 g_cdw_/L of biomass was reached. The following addition of 300 g of methanol, with a volumetric flow that ranged between 1 and 2 g/L∙h during 24 h of induction, only slightly improved the final biomass titer. Accumulation of methanol (3-6 g/L) was observed in both strains 2-3 h after induction indicating a lower initial consumption rate. This also corresponded to a slight decrease of biomass production in the first hours post-induction. On average a 15% higher concentration of biomass was obtained at the end of the process for *Pichia* pink-prel (Fig. 1).

**Fig. 2:**
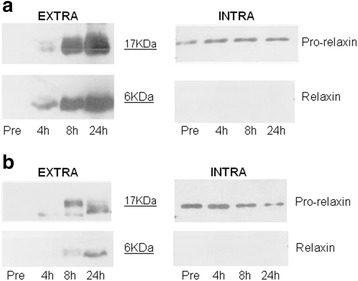
Determination of intracellular and extracellular relaxin and pro-relaxin distribution. Western blotting with anti-prorelaxin anti-relaxin antibodies on intracellular and extracellular fractions collected at different time points during fermentation experiments performed with recombinant strains *Pichia* pink-prel (**a**) and *Pichia* pink-prelHis (**b**)

Samples for pro-relaxin analyses by western blotting were taken during the process for both strains, as described in the methods section. Western blotting experiments with anti-prorel and anti-rel antibodies on cell-free supernatant and cell lysates were performed for both strains at time zero, 4, 8 and 24 h after induction demonstrating different protein patterns in the two strains (Fig. 2). The time zero sample was taken when cells were growing in fed-batch on limiting amounts of glycerol, prior to the addition of methanol. Densitometric analyses of the films indicated an average concentration of 10 and 14 mg/L of pro-relaxin in the fermentation broth 24 h post induction in *Pichia* pink-prel and *Pichia* pink-prelHis, respectively. For both strains the portion of intracellular protein was on average only 1-2% of the total produced.

### Purification of pro-relaxin

**Fig. 3:**
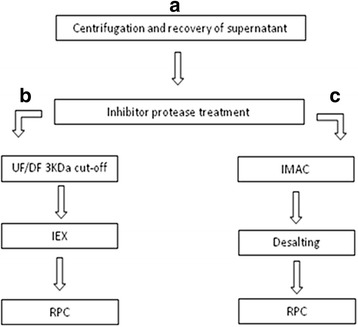
Schematic overview of downstream processes. Initial step used to recover the supernatant from all dual-phase fed-batch fermentation broths (**a**) followed by different strategies used for the purification of pro-relaxin (**b**) and His tagged pro-relaxin (**c**)

**Fig. 4:**
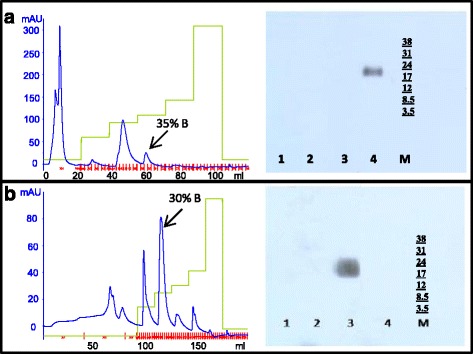
FPLC and Western blot analyses of partially purified recombinant un-tagged and tagged prorelaxin. (**a**) Human recombinant pro-relaxin H2 was purified from fermentation supernatant by ultrafiltration, IEX and RPC. The panel shows the RP chromatogram; the recovered fractions were analysed by western blotting. Lane 1) Unbound, lane 2) 20% B, lane 3) 30% B, lane 4) 35% B, M) molecular weight marker. (**b**) Human recombinant His tagged pro-relaxin H2 was recovered by affinity chromatography in batch and sequentially the sample was loaded on a RPC column; the resulting chromatogram is shown in the panel. The fractions recovered from RPC were analysed by western blotting. Lane 1) Unbound, lane 2) 20% B, Lane 3) 30% B, Lane 4) 35% B, M) molecular weight marker. The arrow in the RP chromatograms indicates the peaks recognized by the anti pro-relaxin antibody

Two different protocols were used for the purification of pro-relaxin from the two recombinant strains (Fig. 3). The first attempt to purify human pro-relaxin H2 from *Pichia* pink-prel was a combination of ultrafiltration and chromatography (ion exchange and reverse phase). The cell free supernatant was initially ultra-filtered on 3 kDa membranes in order to concentrate the sample and then it was loaded on anion exchange column. This step was only useful to partially purify the sample, in fact, separation in the elution phase was not efficient and besides the unbound fraction only one single undefined peak was obtained. The latter was loaded on a RPC column following a gradient step elution, and the recovered fractions were analysed by western blotting (Fig. 4a). The peak eluted with 35% of eluent B corresponds to pro-relaxin and in accordance with IEX results its representativity is quite low. The peak was recovered and analysed by HPLC showing an elution time of about 52.5 min in the set conditions. The samples showing a higher representativeness than 60% were analyzed by mass spectrometry.

The purification of the His tagged pro-relaxin obtained from the fermentation of *Pichia* pink-prelHis did not involve any membrane based step. The fermentation broth was centrifuged and the supernatant was incubated with a Ni FF sepharose resin in batch, in order to attach tagged pro-relaxin. After imidazole separation the sample was loaded on a RPC column that showed the presence of different peaks demonstrating a low representativeness of pro-relaxin (Fig. 4b). As confirmed by western blotting experiments pro-relaxin was only present in the fraction eluted with 30% eluent B, indicating a modification of the interaction of tagged pro-relaxin with the matrix (Fig. 4b). The fraction demonstrating the presence of pro-relaxin was analysed by HPLC and, as previously observed during FPLC purifcation, elution time in these conditions was slightly different, in fact, tagged pro-relaxin was eluted after 56.1 min, probably due to the presence of the His tag and of the Xa factor cleavage site. The pro-relaxin peak area shows a representativeness that is higher compared to that obtained with the recombinant native form, reaching 80% purity.

**Fig. 5:**
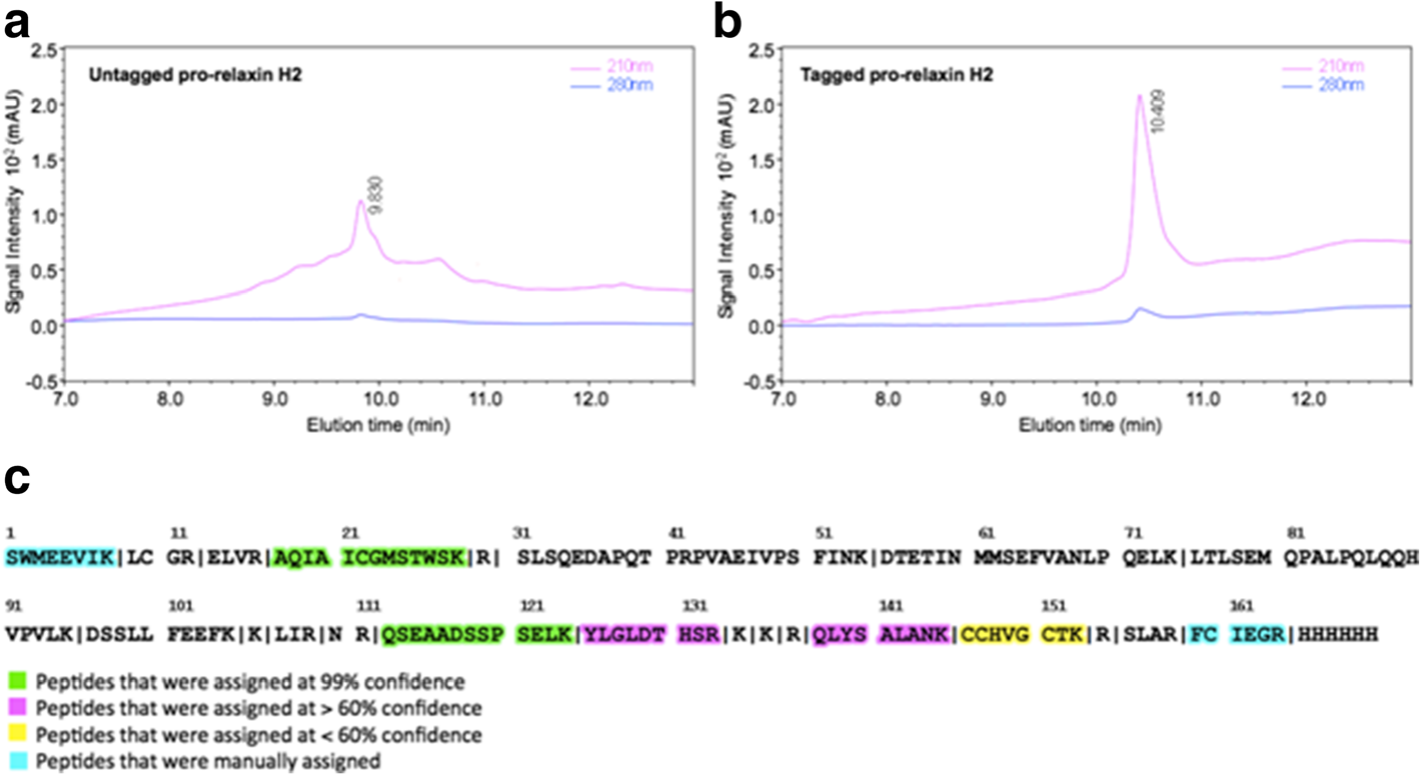
LC-MS/MS analysis of recombinant pro-relaxins. (**a**) RP-HPLC chromatogram with UV detection at 210 and 280 nm of untagged pro-relaxin. (**b**) RP-HPLC chromatogram with UV detection at 210 and 280 nm of tagged pro-relaxin. (**c**) Tryptic digested peptides and sequence covered of tagged pro-relaxin, as assessed by MASCOT and manual analysis of MS/MS data

The purified fractions of both un-tagged and tagged pro-relaxins were analysed by RP-HPLC/ESI-IT-TOF. Figures 5a and b show the analytical chromatogram of the native and tagged pro-relaxins, respectively. Inspection of the two chromatograms clearly shows the different quality of the two purified samples. In the first chromatogram, a broad main peak at R_t_ 9.83 min (53% of acetonitrile - 0.05% TFA) is clearly evident, while the second chromatogram shows a more intense and sharper peak at R_t_ 10.41 min (57% of acetonitrile 0.05% TFA), thus evidencing the higher purity of the tagged pro-relaxin.

The mass spectra of the two samples display several signals indicative of high molecular weight species; however, they were not easily assignable, due to low signal/noise ratio, caused by inefficient ionization of the intact proteins. Therefore, the tagged pro-relaxin, which demonstrated to be the purest sample, was identified by standard proteomic procedures. LC–MS/MS of the tryptic digest and database search in the human subset of NCBI with online available MASCOT software allowed us to confirm the presence of the intact tagged pro-relaxin. In fact, eight peptides were identified, covering 38% of expressed sequence and, most notably, comprising the N- and C-terminal portions (see Fig. 5c). Given the different glycosylation pattern between human and yeast, MS spectra of the identified peptides were checked for O-glycosylation. Post-translational modifications were absent in the seven peptides containing Ser/Thr residues (out of the eight identified). These analyzed peptides contain 50% of the Ser/Thr residues present in the whole sequence.

### EIA immunoassay

**Fig. 6:**
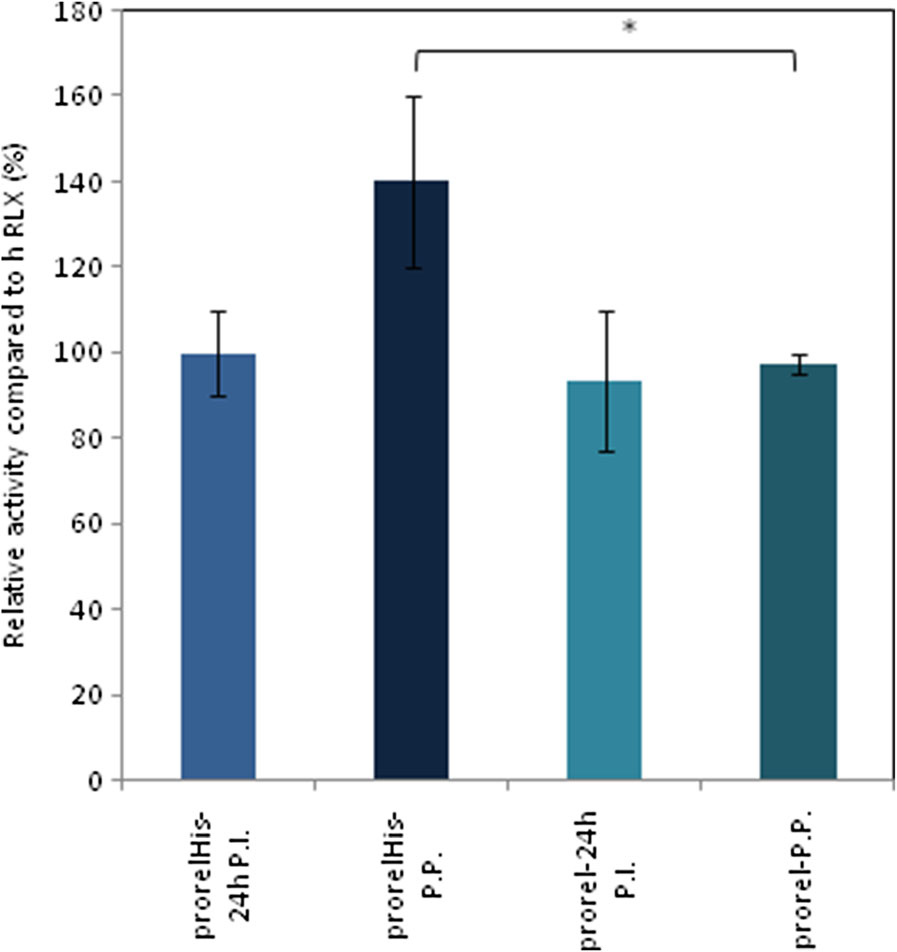
Immunoenzymatic activity assays. Ability of human recombinant pro-relaxin H2 to stimulate cAMP production in a relaxin receptor expressing cell line (THP1) in comparison to the relaxin standard. The tagged and native recombinant pro-forms of the hormone were compared. 24 h P.I indicates that the test was performed on the supernatant collected during the fermentation process; P.P. indicates that the test was performed on prorelaxin samples recovered after purification. Data are mean ± S.D. values of three independent experiments. T-test results are indicated in the figure: *p < 0.05

cAMP levels in the fermentation samples and in the fractions collected after the last purification step were determined by cAMP biotrak EIA assay. The cAMP response of the THP-1 cells were used to test the biological activity of pro-relaxin produced from engineered *Pichia* strains. All fractions of tagged and untagged pro-relaxin resulted active when compared to human relaxin used as control; however, while the supernatant recovered 24 h post induction showed a similar activity in both recombinant strains, samples deriving from the final steps of the purification processes demonstrated a different activity. In particular, a significantly higher (*p* < 0.05) activity was found for the His-tagged pro-relaxin, as compared to the native form (Fig. 6).

## Discussion

Relaxin is a natural hormone with several potential applications the use of which is partly limited by the insufficient and hazardous animal derived protein supply. For this reason numerous attempts to develop a biotechnological process for relaxin production in prokaryotic and eukaryotic hosts were previously evaluated. Porcine pro-relaxin was also expressed in transgenic tobacco [18]. *In vitro* studies demonstrate that porcine relaxin may possess various therapeutic effects, and tobacco was selected because it is a non-food, non-feed crop, and recombinant protein production could readily be scaled up. The authors verified the occurred insertion, transcription and translation of the gene of interest but no concentration or purity data were reported [18]. Also immortalized bovine mammary epithelial (MAC-T) cells were used as hosts for the production of a recombinant tagged equine pro-relaxin by Neumann and colleagues [16]. Cells were transfected with the pST-eRLX construct by using a liposome-mediated method. The concentration of relaxin found in the supernatants was of 0.59 ± 0.22 mg/L while sample lysates showed a concentration of 0.37 ± 0.22 mg/L. The use of MAC-T cells as expression hosts offers the advantage of producing well folded proteins with a correct glycosylation pattern, however, some issues concern process scale-up and the complexity of the medium to be used. *E.coli* was used as host for the expression of both porcine pro-relaxin H2 and of human recombinant relaxin H2 [15, 17]. However, since the proteins were expressed as inclusion body, cell lysis and *in vitro* renaturation were necessary for protein recovery, thereby making the process expensive and not competitive for production scale up. Nevertheless, recombinant human relaxin H2 was the only one that reached phase III clinical trials for patients with acute decompensated heart failure [19].

The aim of the present work was to produce the unprocessed form of this hormone, pro-relaxin, that was shown to posses activity before processing, as well [10, 11]. We chose the metylotrophic yeast *P. pastoris* as expression host*,* since yeasts combine the capability of a protein processing apparatus, that is typical for eukaryotic organisms, to the absence of endotoxins and viral DNA. The human pro-relaxin H2 gene, with and without a C-terminal tag of histidines, was introduced in the genome of a protease deleted strain (Invitrogen, Carlsbad, CA) under the control of the AOX1 methanol-inducible promoter and in frame with a secretion signal, resulting in strains *Pichia* pink-prelHis and *Pichia* pink-prel, respectively. Fed-batch processes based on a dual phase growth on glycerol and methanol were performed on 22 L fermenters resulting in a final yield of about 35 g_cdw_/L of biomass. One of the main problems commonly encountered during recombinant protein production is protein loss due to protease degradation. We therefore modified the protocol suggested by Pai-Chanfrau and collegues [20] that evaluated the effect of temperature, pH and addition of excess nitrogen source and EDTA, on mini-pro-insulin expression. It was also previously observed that addition of casaminaocids reduced proteolysis of ovin interferon in *P. pastoris* by acting as preferential substrates [21]. We used a medium containing casein hydrolysate as main N source, and, before methanol induction, one single pulse of EDTA and ammonium sulphate was performed to divert proteases activity. The behavior of the recombinant strains was quite similar in terms of final biomass, growth rate and C-source consumption rate. Although *Pichia* is one of the hosts that is most commonly used for protein secretion several studies aim to improve the secretion of foreign proteins due to the variability of efficiency [22, 23]. In both strains 1-2% of pro-relaxin could also be found inside the cells indicating a partial, although almost negligible, failure of the secretion system. Expression of secreted tagged pro-relaxin strongly increased during induction whereas it remained constant inside the cell. Moreover the processed form of the pro-hormone (relaxin) was detected in both strains in the extracellular fraction. One of the key steps involved in secretion and activation of peptide hormones is site specific proteolysis, which is mediated in eukaryotic cells by serine proteases. In yeast the Kex2 enzyme is undertaking this role. It was previously demonstrated that Kex2 mainly targets proteins that are secreted to the environment [24] and coexpression of human pro-relaxin with Kex2 from *Saccharomyces cerevisiae* in human kidney cells resulted in the secretion of mature relaxin [25]. It was therefore not surprising to observe relaxin in the extracellular fraction of broth samples. The result is a potentially more powerful active principle composed of a mixed population of pro-relaxin, that should have a higher half life and therefore a better pharmacokinetic performance, and relaxin, for immediate applications. Western blot densitometric analyses performed on the supernatants and cell lysates showed quite similar concentrations of native and tagged pro-relaxin at the end of the fermentation process, that ranged between about 10 and 14 mg/L, respectively.

Different procedures were used for the purification of the native and tagged forms of pro-relaxin produced. However, one of the major issues encountered during downstream processing was protein degradation. The output of the computational analysis of the human pro-insulin aminoacidic sequence with the ProtParam tool [26] indicates an instability index of 37.52 and classifies the protein as “stable”. The same analysis performed on pro-relaxin H2, that also belongs to the insulin-like family of hormones, results, on the contrary, in an instability index of 77.46 and classifies the protein as “unstable”.

Both purification strategies allowed the attainment of proteins with the expected aminoacidic sequence and that also demonstrate binding to the relaxin receptor and therefore imply biological activity compared to the human relaxin control. A comparison of the purification efficiency and yield obtained during the two processes is difficult to perform due to the absence of a method that allows a precise determination of the concentration of pro-relaxin in the fermentation broth and after each purification step. The first strategy used for native pro-relaxin purification, based on a sequence of UF, IEX and RPC chromatography resulted in a protein with a final purity of 60% as determined by HPLC. The difficulties found during the purification procedure addressed us towards the development of a second strategy focused on the addition of a 6xHis-tag in order to (i) reduce protein losses due to the interaction with UF membranes (ii) minimize degradation by reducing process time (iii) perform most steps at low temperature in controlled conditions. The tag was added at the C-terminus of human pro-relaxin. Moreover a Xa factor cleavage site was inserted after the tag to allow removal of the latter after the purification step. The C-terminus was chosen, based on the work that investigated on the geometry of the relaxin receptor binding site, according to which addition to the C-terminus would not interfere with residues defined as critical for receptor binding, on the B chain interface [27]. This approach resulted in a final sample purity of up to 80%.

The higher content of intact pro-relaxin in the tagged sample was confirmed by LC-MS analyses. In fact, the RP-HPLC profiles clearly indicate the higher recovery and purity of the tagged purified fractions. This allowed a complete proteomic analysis of the tryptic digest, confirming the intact sequence of the tagged recombinant protein. Thus, LC-MS/MS analysis with IT-TOF platform revealed a valuable tool for rapid and sensitive analyses, which can be used in combination with biochemical methodologies, in order to confirm the purity and the chemical structure even in complex samples, with the ultimate purpose of explaining differences in functional properties [28, 29]. Our spectra analyses also excluded any post-translational modification. This is particularly meaningful, since hypermannosylation is a known issue in yeast expression. In fact, hypermannosylated proteins may imply drawbacks, such as allergic reactions, thus limiting their therapeutic use [30, 31].

## Conclusions

With the aim of developing an alternative to currently used extraction procedures from animal tissues and possibly prokaryotic relaxin production avoiding recovery from inclusion bodies, the present study demonstrates the possibility of producing biologically active human pro-relaxin and relaxin by exploiting the yeast *P. pastoris*. The purified recombinant proteins underwent a complete proteomic analysis confirming their intact sequence with a greater purity of the tagged protein. Optimization of the fermentation strategy and of the purification process are however necessary to improve biomass titers for pharmaceutical applications.

## Methods

### Materials

Genomic DNA and plasmid DNA were isolated using Qiagen DNeasy kit and Qiagen miniprep kitt (Qiagen, Valencia, CA) respectively according to the manufacturer’s instructions. Restriction endonuclease digestions, DNA ligations, SDS-PAGE and agarose gel electrophoresis were performed using standard techniques [32]. LCMS ultragradient grade water, acetonitrile and formic acid were supplied by Romil, Cambridge, UK. TFA was purchased from Applied Biosystems (Carlsbad, California). Proteomic grade trypsin, dithiothreitol (DTT) and iodoacetamide (IAA) were supplied by SIGMA.

### Construction of *Pichia pastoris* expressing human pro-relaxin H2

The sequence coding for human pro-relaxin isoform H2 was synthesized by MWG (Milan, Italy) and the codon usage was optimized for expression in *P. pastoris*. The optimized sequence is reported in additional file 1. The gene was amplified with ProrlxUp:5’ –TCTTggATggAAgAggTgATcAA-3’ and either ProrlxDw: 5’ ggTACC TTAACAAAACCTAgCAAggg 3’ or ProrlxHISDw: 5’ ggTACC tta atgatgatgatgatgatgtctaccctcaat ACA AAA CCT AgC AAg gg 3’ for the insertion of the C-terminal histidine tag. Both reverse primers contain the *Kpn*I recognition site for cloning into pPinkα-HC (Invitrogen, Carlsbad, CA). This integration vector contains the alpha mating pre-sequence necessary to guide protein secretion and the methanol inducible promoter AOX1. The recombinant vectors were sequenced by BMR genomics (Padoa, Italy) to verify sequence accuracy and in frame insertion of pro-relaxin with the secretion sequence.

Transformation was performed according to Invitrogen guidelines. The pPinkα-prorlxPp and pPinkα-prorlxhisPp recombinant vectors were linearized with *Pme*I before electroporation into *Pichia* pink strain 4 (Invitrogen, Carlsbad, CA). The strain is a double knock-out for two vacuolar proteases encoded by *pep4* and *prb1*. Positive clones were selected on adenine drop out medium, and the presence of the pro-relaxin gene was further verified by colony PCR.

### Shake flask experiments

Positive clones were screened by inoculating single colonies in 10 ml of BMGY medium containing glycerol 10 g/L, yeast extract 10 g/L, bactopeptone 20 g/L, potassium phosphate buffer pH = 6.0 100 mM, biotin 0.0004% p/v, yeast nitrogen base 1.34% p/v in 125 ml shake flasks. Colonies were grown in agitation at 30 °C for 24 h. Cells were then collected by centrifugation and resuspended in BMMY that contains 0.5% v/v methanol instead of glycerol to induce recombinant protein expression. Pulses of 0.5% v/v of pure methanol were performed after 24 h and 48 h for a total of 72 h of induction. Supernatants were collected to verify and compare the presence of the protein in the different clones. The supernatants were concentrated and diafiltered on 50 and 3 KDa ultrafiltration membranes and analysed by western blotting.

### 22-L fermentation experiments

Fermentation experiments were carried out in a Biostat C reactor (Sartorius Stedim; Melsungen, Germany) with initial working volume of 7 L. The medium used for all experiments modified from [20] contained: 20 g/L glycerol, 10 g/L casein hydrolysate, 13.4 g/L YNB, 0.0004 g/L biotin and 100 mM KH_2_PO_4_ K_2_HPO_4_ salts. Temperature was set at 22 °C, pH was fixed at 6.3 *via* automated addition of 30% v/v NH_4_OH and 30% v/v H_3_PO_4_. The airflow was kept constant at 1.4 vvm. The recombinant strains were pre-cultured in shakeflasks at 22 °C for about 21 ± 1 h in 200 ml of medium. The culture was transferred to the bioreactor and after approximately 24 ± 2 h the sharp increase of the DO peak indicated the depletion of the initial carbon source. The starting concentration of glycerol and casein were restored by the addition of a concentrated medium (280 g/L glycerol, 140 g/L casein) the consumption of which was followed by a glycerol DO-stat phase (PO_2_ set point 30%) for a total of about 43 ± 2 h. In order to maintain an excess of nitrogen source before initiating the methanol induction phase about 0.03 M EDTA and 0.26 M ammonium sulphate were also added. An exponential feeding profile ranging from 1 to 1.4 g/L∙h of methanol in the first 8 h after induction, and from 1.5 to 2 g/L∙h from 8 to 24 h post induction was set, and cultures were stopped after 24 h.

For the duration of all cultivations broth samples were withdrawn from the reactors at regular time intervals for the determination of substrates and extracellular metabolites.

Broth samples were also withdrawn pre-induction and 4, 8 and 24 h post-induction with methanol for western blot analyses to compare the amount of intracellular and secreted pro-relaxin over time.

Fermentation experiments were performed in triplicate for each strain, and curves showing biomass production and, glucose and methanol consumption are mean values ± S.D. for each time point.

### Downstream processes

The culture broth was centrifuged at the end of the fermentation processes at 5000xg at 4 °C for 30 min (Avanti J26 XP, Beckman CoulterTM) and the supernatant was recovered. Ten microliters/L of protease inhibitor (Complete protease inhibitor, Roche) prepared by dissolving one table in 10 mL of ultrapure water was immediately added to the supernatant at a concentration of 100 μl/L. Figure 3 shows the purification protocol used for tagged and untagged pro-relaxin. The clarified supernatant was concentrated by tangential flow filtration (TFF) on a Sartoflow alpha system (Sartorius Stedim, Milan, Italy). The cell-free supernatant was ultrafiltered using 3-kDa poliethersulfone membranes with a filtering area of 0.5 m^2^. The feed flow rate and TMP were kept constant at 1 L/min and 0.3-0.6 bar, respectively, during the process. The final concentration factor of the sample was 10 and the DF exchange factor 2. Ultrapure water (MilliQ; Millipore, USA) was used as DF buffer.

Two different strategies were used at this point for the purification of tagged and un-tagged pro-relaxin as described in the figure (Fig. 3). The clarified supernatant was concentrated by Tangential flow filtration (TFF) using a Sartoflow system (Sartorius Stedim, Milan, Italy). Polyethersuphone cassettes, having a cut-off of 3 kDa, were used to reduce permeation of pro-relaxin (18 kDa) and eventually relaxin (6 kDa). The filtering area used was 0.07-0.1 m^2^ per liter of supernatant. During the process, feed flow rate and transmembrane pressure (TMP) were constant at 1 L/min and 0.3-0.6 bar, respectively. The final concentration factor of the sample was 13 ± 2 . Supernatant was diafiltered twice with ultrapure water to reduce sample conductivity before being loaded onto an ion exchange chromatography.

### Ion metal affinity chromatography (IMAC)

Ten ml of IMAC resin (Ni FF sepharose 6 fast flow, GE Healthcare, Milan, Italy) were added for every liter of sample and incubated in batch at 300 rpm and 4 °C for 3 h. The samples were centrifuged at 5000xg and 4 °C for 20 min (Avanti J26 XP, Beckman CoulterTM), and in order to remove aspecifically bound proteins, the resin was washed, for 15 min at 300 rpm, with a washing buffer containing 20 mM imidazole, 500 mM sodium chloride, pH 7.4 in the ratio of 1.5:1 with the resin. This step was performed twice. The tagged protein was recovered by suspending the resin in 500 mM imidazole, 500 mM sodium chloride, pH 7.4 (1.5:1) at 300 rpm at 4 °C for 20 min [33].

### Desalting

Fractions eluted from IMAC were loaded onto a desalting column (HiPrep 26/10 desalting, GE Healthcare, Milan, Italy). In order to reduce salt concentration and remove imidazole, proteins were eluted with ultrapure water (MilliQ; Millipore, USA) in 1.5 column volumes (CV) at a flow rate of 1 ml/min. Eluted proteins were detected at 214 and 280 nm and then separated by reverse phase chromatography (RPC).

### Reverse phase chromatography

A preparative reverse-phase column (RPC Resource 3 ml, GE Healthcare, Milan, Italy) previously equilibrated with 95% buffer A (water, 0.1% trifluoracetic acid) and 5% buffer B (acetonitrile, 0.1% trifluoroacetic acid) was used for sample purification. A multistep gradient (5 CV of 20% B, 5 CV of 30% B, 5 CV of 35% B, 5 CV of 45% B, 5 CV of 95% B) of buffer B at a flow rate of 1 mL/min was used to separate peptides into different 2 mL fractions. Eluted proteins were detected at 214 and 280 nm. All fractions collected were freeze dried (Epsilon 2-6D, Martin Christ, Germany) and analysed by western blotting and LC-MS.

### Ion exchange chromatography (IEX)

The concentrated and diafiltered retentate containing pro-relaxin and other host proteins was loaded with buffer A (Sodium Acetate 20 mM, Sodium Chloride 5.0 mM, pH 5.5) on an anion exchange column (HiPrep CM FF 16/10, GE Healthcare, Milan, Italy. Proteins bound to the column were eluted applying a linear salt gradient ranging from buffer A to buffer B in 5 column volumes at flow rate of 1 ml/min and collected in 2 ml fractions. Eluted proteins were detected at 214 and 280 nm. Salts were washed on 3KDa ultra filtering devices (amicon,Millipore, Milan, Italy) lyophilised (Epsilon 2-6D, Martin Christ, Germany) and analysed by western blotting. All fractions were further purified through a reverse phase chromatography (RPC).

### HPLC analyses

Fractions obtained throughout the purification processes were analyzed by RP-HPLC. The system used was a Dionex Ultimate 3000 (Thermofisher, Milan, Italy) equipped of UV and RI detectors. The samples were loaded with buffer A (water, 0.1% trifluoracetic acid) and applied to a Vydac C8 4.6 x 150 mm, 5 μm (Grace, USA) column temperature controlled at 25 °C C. Proteins bound to the column were eluted applying a linear gradient from 5 to 40% of buffer B (acetonitrile, 0.1% trifluoroacetic acid) in 50 min at flow rate of 0.5 ml/min. Eluted proteins were detected at 280 nm.

### RP-HPLC/IT-TOF mass spectrometry, LC-MS data acquisition

LC-MS analyses were performed using an LC-MS/ESI-IT-TOF instrument (Shimadzu Europe, Manchester, UK). All the spectra were acquired using the LCMS Solution software. Mass calibration and tuning were performed according to the manufacturer’s recommendations.

Each sample of intact un-tagged and tagged pro-relaxin H2 (0.4 μl injection volume), dissolved in aqueous solution, was injected onto the LC-MS at a flow rate of 0.2 ml/min. The analyses were performed using an analytical Vydac (Hesperia, CA) C18 column (2.1 x 100 mm, 5 μm), with water and acetonitrile (0.05% TFA) as eluents. The proteins were eluted with a linear gradient of acetonitrile- 0.05% TFA, 15 - 65% over 8.5 min. Mass spectra were acquired in positive scan mode over m/z 600 - 2000 at 30 msec ion accumulation time. An electrospray voltage of 1.60 kV and a nitrogen gas flow of 1.5 L/min were employed.

Enzymatic digestion of the tagged protein was carried out with 40 pmol of proteomic grade trypsin, in 25 mM NH_4_HCO_3_ buffer, pH 7.5. Samples were incubated for 15 min with 10 mM DTT at 50 °C, and for 15 min with 20 mM IAA at room temperature prior to digestion, then trypsin solution was added and samples were incubated overnight at 37 °C. Peptide digest was pretreated prior to LC-MS/MS analysis via solid phase extraction tecnique (C18-Supelco) to remove detergents. Sample of the peptide digest (2 μl injection volume), dissolved in aqueous solution, was injected onto the LCMS at a flow rate of 0.3 ml/min. The analyses were performed using an analytical VydacProzap MS C18 column (2.1 x 20 mm, 1.5 μm), with water and acetonitrile (0.1% formic acid) as eluents. The peptides were eluted with a linear gradient of acetonitrile - 0.1% formic acid, 5 - 100% over 14 min. Mass spectra were acquired using automatic acquisition mode, consisting in one MS scan (mass range from 350 to 2000 m/z) followed by MS/MS scans of the most abundant ions in each MS scan. MS/MS spectra were measured automatically when the MS signal surpassed the threshold of 50,000 counts, at 50 msec ion accumulation time. Each LC-MS/MS analysis was preceded and followed by blank runs to avoid carryover contamination.

### MS/MS data analysis

The acquired MS/MS spectra were transformed in Mascot Generic format (.mgf) and used to query the NCBI database 20151109 (76,068,736 sequences), with taxonomy restriction to Human (314,559 sequences), for protein identification with an online open access version of MASCOT software (www.matrixscience.com). Mascot search parameters were: trypsin as enzyme; 3, as allowed number of missed cleavages; carbamidomethylation of cysteines as fixed modification; 20 ppm MS tolerance and 0.6 Da MS/MS tolerance; peptide charge from +1 to +3. Possible oxidation of methionine and the formation of pyroglutamic acid from glutamine residues at the N-terminal position of peptides were considered as variable modifications. Since NCBI Entry does not correspond exactly to the expressed sequence, we manually searched for N- and C-termini peptide fragments. We were able to find the 1-8 and 159-164 peptides, further confirmed by their MS/MS spectra.

### Cell lysis

Five grams of wet biomass were suspended in 25 ml of lysis buffer containing 50 mM sodium phosphate, 1 mM EDTA and 5% glycerol. Glass beads were added to the suspension and vortexed at 4 °C for 1 h. The supernatant was treated with 50 U/ml of Dnase I for 40 min at 4 °C and microfiltered on 0.45 μm filters.

### Western blotting

Standard western blot protocols were used to monitor the progress of the purification and qualitative evaluation of the samples. The protein concentrations were determined using the Bio-Rad protein assay reagent (Bio-Rad Laboratories, Milan Italy). Whole cell extracts and equal amounts of proteins were fractionated by SDS-PAGE and transferred to a nitrocellulose membrane using a transfer apparatus according to the manufacturer’s protocols (Bio-Rad Laboratories, Milan Italy). After incubation with 5% w/v skimmed milk in Tris-buffered saline (10 mM Tris, pH 8.0, 150 mM NaCl, 0.5% Tween 20) for 60 min, the membrane was washed once with Tris-buffered saline (TBST) and incubated with antibodies against Pro-relaxin Immunodiagnostik (1:400), and Relaxin R&D (1:250) at room temperature for 2 h. Membranes were washed three times for 10 min and incubated with a 1:10000 dilution of horseradish peroxidase-conjugated anti-mouse and anti-rat antibodies for 1 h. Blots were washed with TBST three times and developed with the ECL system (Amersham Biosciences) according to the manufacturer’s protocols. The protein concentration was quantified using a protein assay kit from Bio Rad Laboratories (Hercules, CA, USA) according to the manufacturer’s instructions. Densitometric analysis of the film was performed using a Model Universal Hood II imaging densitometer (Bio-rad Laboratories) in trasmittance mode and analysed using Bio-Rad discovery software. Bands were normalised on the relaxin pure standard at different concentrations (0.5-1-2-5 mg/ml)

### Pro-relaxin *in vitro* bioassay

Recombinant pro-relaxin H2 was assayed for its ability to induce cAMP production in a relaxin-expressing cell line (THP-1) [34]. THP-1 cells (ATCC, LGC Standards Italy) were grown in DMEM-F-12 containing 10% (v/v) FBS, 1% (v/v) penicillin–streptomycin. The cells (5.0x10^4^ cells/well) were cultured overnight in a 96-well culture plate and were incubated at 37 °C for 30 min with varying concentrations (range 10^−6^ to 10^−9^M) of relaxin (recombinant-human-relaxin) used as reference and recombinant pro-relaxin H2 samples in the presence of 1 mM forskolin and 50 mM isobutylmethylxanthine (IBMX). The plate was then briefly centrifuged, the medium was removed and the cells were resuspended in lysis buffer. cAMP levels in cell lysates were determined using Amersham cAMP Biotrak enzyme immunoassay system (VWR s.r.l, Italy). cAMP was evaluated in the range of 12.5 to 3200 fmol/well generating a standard curve by plotting the B/B_0_ percentage as a function of the log cAMP concentration. The cAMP production was normalised, for each concentration tested, on that obtained with a positive control of human relaxin of known concentration. The results are presented as average relative responses.

Each sample (fermentation supernatant or post-purification) was analysed by performing three independent experiments (3 assay plates) each in triplicate, and results are indicated as mean ± S.D. for each sample. Statistical significance was evaluated by T-student test.

## Additional file

Additional file 1: Codon optimized DNA and aminoacidic sequence of human pro-relaxin H2. (DOCX 11 kb)

## Abbreviations

DO: Dissolved oxygen
IEX: Ion exchange chromatography
RPC: Reverse phase chromatography
UF: Ultrafiltration
