# Supplementary material for: Production of human pro-relaxin H2 in the yeast Pichia pastoris
Source: BMC Biotechnol. 2017 Jan 14;17:4. doi: 10.1186/s12896-016-0319-0 (PMC5237503; doi:10.1186/s12896-016-0319-0)
Supplement: Additional file 1: — Codon optimized DNA and aminoacidic sequence of human pro-relaxin H2. (DOCX 11 kb) [file 12896_2016_319_MOESM1_ESM.docx]

DNA (A) and aminoacidic (B) sequences of Human prorelaxin H2 codon optimized for expression in *Pichia pastoris.*

Red indicates Xa factor recognition site and green indicates the His tag

(A)

tcttggatggaagaggtgatcaaattgtgtggtagagaactggtaagagcacagatagccatttgcggtatgtctacttggagcaaacgtagcctttctcaggaagatgctcctcaaactccaagaccagttgccgaaattgttccttccttcattaacaaggacacagagaccatcaacatgatgtccgaatttgtcgccaatttgccccaagagttgaaactcaccttgtcagaaatgcaacctgctttaccgcagttacaacaacatgtgccagtcttgaaggactcttcactactgtttgaggagttcaagaagctgattcgtaatcgacaaagtgaagctgctgatagttcaccatctgagctcaagtacttaggcttggatactcactcgagaaagaaaagacagctttatagtgcactagctaacaaatgctgtcatgttggatgtacgaaaaga t

cc ctt gct agg ttt tgt att gag ggt aga cat cat cat cat cat cat taa

(B)

SWMEEVIKLCGRELVRAQIAICGMSTWSKRSLSQEDAPQTPRPVAEIVPSFINKDTETINMMSEFVANLPQELKLTLSEMQPALPQLQQHVPVLKDSSLLFEEFKKLIRNRQSEAADSSPSELKYLGLDTHSRKKRQLYSALANKCCHVGCTKRSLARFCIEGRHHHHHH
